# Inferring social signals from the eyes in male schizophrenia

**DOI:** 10.1038/s41537-024-00527-4

**Published:** 2024-11-14

**Authors:** Annika Resch, Jonas Moosavi, Alexander N. Sokolov, Patrick Steinwand, Erika Wagner, Andreas J. Fallgatter, Marina A. Pavlova

**Affiliations:** 1 Department of Psychiatry and Psychotherapy, Tübingen Center for Mental Health (TüCMH), Medical School and University Hospital, Eberhard Karls University of Tübingen, Tübingen, Germany; 2 German Center for Mental Health (DZPG), Partner Site Tübingen, Tübingen, Germany

**Keywords:** Schizophrenia, Emotion

## Abstract

Nonverbal communication habitually leaks out in ways that expose underlying thoughts, true feelings, and integrity of a counterpart. Social cognition is deficient in a wide range of mental disorders, including schizophrenia (SZ). Inferring social signals through the eyes is pivotal for social interaction but remains poorly investigated. The present work aims to fill this gap by examining whether and, if so, how reading language of the eyes is altered in SZ. We focused on male SZ, primarily because the disorder manifests a gender-specific profile. Patients and matched typically developing (TD) individuals were administered the Reading the Mind in the Eyes Test-Modified (RMET-M) and Emotions in Masked Faces (EMF) task that provide comparable visual information. The findings indicate that in SZ, the emotion recognition profile is similar to TD, with a more accurate recognition of some emotions such as fear, neutral expressions, and happiness than the others (sadness and disgust). In SZ, however, this profile is shifted down: all emotions are recognized less accurately than in TD. On the RMET-M, patients are also less precise, albeit they perform better on items with positive valence. In SZ only, recognition accuracy on both tasks is tightly linked to each other. The outcome reveals global challenges for males with SZ in inferring social information in the eyes and calls for remediation programs to shape social cognition. This work offers novel insights into the profiles of social cognitive deficits in mental disorders that differ in their gender prevalence.

## Introduction

Nonverbal communication habitually leaks out in ways that expose underlying thoughts, true feelings, and credibility of a counterpart, providing insights that may contradict a verbal information flow that is believed to be more easily kept under control and, therefore, in most cases less fluid and reliable^1–4^. The ability to infer emotions and drives of others is often altered in individuals with mental disorders such as schizophrenia (SZ)^5–19^ preventing efficient social interaction.

SZ represents a chronic mental disorder that affects one in 300 people worldwide^20^. It has the highest prevalence among psychotic mental disorders^21^ and is associated with a reduced life expectancy of almost 15 years compared to the general population^22^. Along with positive symptoms such as hallucinations and delusions^21,23^, and negative symptoms such as alogia, avolition, and asociality^24^, hitches in social cognition is one of the core factors determining the quality of life in individuals with SZ. Inefficient or even maladaptive social cognition can lead to difficulties in establishing and maintaining relationships and to reduced social support having an impact on the functional outcome, in particular, in vocational domain^6^. Deficits in social functioning often start before the onset of psychotic symptoms and persist throughout the whole course of the mental disorder^9,25^. Profound impairments in inferring emotions have been found in body language reading^26,27^ (for reviews, see refs. ^8,28,29^) and in processing of facial information^10,11,30–32^, including reading emotions in the eyes, often referred to as *the windows to the soul*.

The Reading the Mind in the Eyes Test (RMET)^33^ is widely used as a valuable tool for assessing nonverbal social cognition in typical and atypical development^3,17,34–36^. Patients with SZ are reported to take longer and be less accurate on the RMET than their typically developing (TD) peers^34,37–43^. In SZ, meta-regression analysis found a negative association of RMET score with age and a positive association with years of schooling^17^. Only few studies examined whether inferring mental states on the RMET depends on valence of expressions (positive, neutral, and negative), and reported that individuals with SZ perform worse than controls on negative and neutral items^44,45^.

For social cognition, an overall amount of information provided by the RMET images is comparable to that offered by faces covered by masks^36,46^. In accord with this, performance on the RMET was found to predict accuracy of facial affect recognition in masked faces^47^. Plentiful studies elicited by the COVID-19 pandemic regulations with obligatory mask wearing reveal a specific pattern in reading emotions in the eyes, with the uneven impact of masks on distinct emotions. Inferring sadness and disgust are reported to be most inaccurate^48–69^. However, for other emotions such as neutral expressions, fear, and happiness, visual information from the eyes is sufficient for efficient recognition^48–50,56,57,59,61,62,65,67–71^.

How challenging is inferring emotions in masked faces in schizophrenia? Only a handful of studies have addressed this issue, and the outcome is rather inconclusive. In a relatively small and inhomogeneous sample of patients with SZ (*N* = 13, 7 males), face masks substantially hindered recognition of happiness expressed with low (but not with high) intensity^72^.

Reading language of the eyes is thought to be gender-specific. The advantage of women (relatively small in effect size, but persistent) on reading language of the eyes as assessed by the RMET is well-documented^2,73,74^ (for review, see refs. ^36,46^). Yet, the outcome of studies on reading emotions in faces covered by a mask (mostly conducted online with samples predominated by females) is controversial, either pointing to proficiency of females^75,76^ or not^55,77^. By contrast, males are found to be more capable in disgust recognition, which is of special value for a better understanding of mental disorders^71^. On the RMET, female patients with SZ in remission score higher and have a higher empathy level than males^40^.

The present study was aimed at examination of inferring social signals through the eyes in SZ. We concentrated on male SZ because (i) males are affected 1.4 to 1.6 times as often as females^23,78^; (ii) SZ is a gender (a social construct)/sex (a neurobiological one)-specific mental disorder with substantial differences in its manifestation, with male patients exhibiting an earlier age of onset, poorer premorbid social functioning, more severe negative symptoms (especially social withdrawal, blunted or incongruent affect) and a higher rate of substance and alcohol abuse^79–81^; and, most important, (iii) females and males with SZ demonstrate distinct profiles in social cognition and metacognition, with females performing generally better on emotion recognition tasks^12,19,82^.

In the present study, we investigated: (a) whether inferring basic emotions in masked faces (as assessed by the Emotion in Masked Faces, EMF, task) and more complex mental states (as assessed by the RMET) is impaired in male SZ, (b) if so, whether these alterations are global or selective (emotion- and valence-specific), and (c) whether performance on the RMET is linked to reading emotions in masked faces in terms of accuracy and processing speed.

## Methods

### Participants

Fifty-eight participants were enrolled in the study. Twenty-nine patients with SZ were recruited from inpatient units at the Department of Psychiatry and Psychotherapy, University Hospital of Tübingen, Germany. One patient and, accordingly, matched to him TD control were excluded from the final data processing due to alterations in his diagnosis during his inpatient stay. The final sample of 28 patients was aged 31.54 ± 10.57 years (mean ± standard deviation, SD, median, Mdn, 28 years, 95% confidence interval, CI [27.44; 35.64], age range, 18 to 54 years). The sample size was determined a priori to account for potential dropouts during statistical data processing. Eighteen out of 28 patients were diagnosed with paranoid SZ (International Statistical Classification of Diseases and Related Health Problems, 10^th^ Edition, ICD-10, F20.0), one patient with other SZ (F20.8, cenesthopathic SZ), one with schizotypal disorder (F21), and eight patients with schizoaffective disorder [two of them of manic (F25.0), two of depressive (F25.1), and four of mixed type (F25.2)]. The average time from the first diagnosis till examination was 7.12 ± 9.66 years (Mdn, 4 years; 95% CI [3.13;11.11]. Seventeen out of 28 SZ patients had one or more comorbidities such as abuse of nicotine, alcohol and other drugs (Table S1, Supplementary Material). Twenty-seven out of 28 SZ patients were under medication (Table S1, Supplementary Material).

Twenty-eight TD individuals, person-by-person matched with patients with SZ were recruited from the local community. Each TD control was tested after a respective SZ patient had been tested. They matched patients with respect to gender and age (31.64 ± 11.1 years, Mdn, 28 years; 95% CI [27.34;35.94], age range, 21 to 57 years). There was no difference in age between SZ patients and TD controls (Mann-Whitney test, *U* = 386, *p* = 0.928, two-tailed, n.s.). None of them had a history of neurological or psychiatric disorders [including SZ, major depressive disorder (MDD), autism spectrum disorders (ASD), and attention deficit hyperactivity disorder] or regular medication intake.

All participants were native German speakers and had normal or corrected-to-normal vision. Participants were tested individually and received a monetary reward for their participation. The study was conducted in accordance with the Declaration of Helsinki and approved by the local Ethics Committee of the Medical School, University of Tübingen, Germany. Informed written consent was obtained from all participants. Participation was voluntary, and the data was processed anonymously.

### Emotions in Masked Faces (EMF) task

**Fig. 1:**
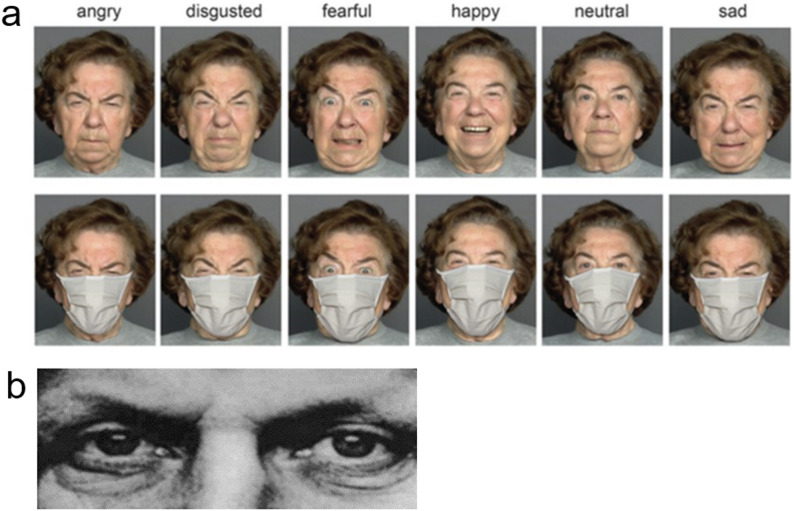
Illustrations of stimuli used. **a** A female poser expressing six basic emotions. Faces are shown under full-face (top) and covered-by-mask conditions (bottom). From Carbon (2020), Front Psychol.^48^, the Creative Commons Attribution [CC BY] license. These images are presented for illustrative purposes only, and have not been used as experimental material here. **b** An example of images used in the RMET. From Baron-Cohen et al. (2001). J Child Psychol Psychiatry^33^. Copyright © 2003 by John Wiley and Sons Inc., reprinted with permission of the publisher.

The Emotion in Masked Faces (EMF) task is described in detail elsewhere^71^. To summarize, frontal photographs of six Caucasians (3 females and 3 males) were used from different age groups (young, middle, and older age). A graphics editor was employed to superimpose a mask on each face. Each person expressed six emotions (anger, disgust, happiness, neutrality, sadness, and fear; Fig. 1). In total, 108 trials, consisting of 36 images (6 emotions × 2 genders × 3 age groups) repeated three times per session, were presented. The photographs were shown in a pseudo-randomized order, one at a time for 2 s in three runs separated by short breaks between them. After stimulus offset, two words (correct and incorrect response) appeared on the left and right side of the screen. By using only two of the original six response options, the difficulty of the task (in the sense of decision-making complexity as well as reliance on language proficiency and comprehension) and test duration (both of which are welcome in examination of patients) were decreased. The response alternative pairs were chosen based on emotion confusion data^48,77^: angry - disgusted, neutral - happy, and sad - fearful. Participants were asked to choose the word that best described the displayed emotion and to respond as accurately and as fast as possible by pressing a key on the side of the correct response. Immediately after response, a white fixation cross appeared in the middle of the screen for an interstimulus interval that lasted for 1.5–2 s. If participants failed to respond within a time limit of 5 s, the next trial started automatically. Participants were administered a computer version of the task by using Presentation software (Neurobehavioral Systems, Inc., Albany, CA, USA). The stimuli subtended a visual angle of 9.8° × 9.8° at an observation distance of 70 cm. The instructions were carefully explained, and each participant had to pass through a short pre-test before the real test began. No immediate feedback regarding performance was given to the participants. On the EMF task, 2.89 ± 5.25 responses were missed in the SZ group (Mdn, 1.00; 95% CI [0.86; 4.93]), and 0.36 ± 0.68 responses were missed in the TD group (Mdn, 0; 95% CI [0.09; 0.62]).

### Reading the Mind in the Eyes Test, modified (RMET-M)

The original version of the RMET is described in detail elsewhere^33^. The present version had been modified in such a way that only 16 monochrome photographs of the eye portion of female and male faces expressing a certain emotion or affective state (instead of 36 in the original version) and two response alternatives (correct and incorrect, instead of four alternative adjectives in the original version) were used^3^. As a result, performance on the RMET-M was less dependent on language comprehension skills and examination time became shorter. The images were selected to compose a balanced set containing an equal number of female/male depictions (eight women and eight men) and positive/negative valence of expressions (eight positive and eight negative). In total, each experimental session consisted of a set of 80 trials (16 photographs × 5 repetitions) presented in a pseudo-randomized order. Each image was exposed for 2 s at an observation distance of 70 cm after which two adjectives (correct and incorrect responses) appeared on the left and right of a black screen. The correct response position varied randomly across trials. Participants were asked to respond as accurately, but also as fast as possible during a time limit of 12 s. After each response, during an interstimulus interval that randomly varied between 2 and 3 s, a white fixation cross was displayed in the center of the screen. If participants failed to respond, the next trial started automatically. On the RMET-M, 0.79 ± 1.55 responses were missed in the SZ group (Mdn, 0; 95% CI [0.19; 1.39]; not more than six by a single patient), while only one miss in one participant occurred in the TD group. Completion of both the EMF task and RMET-M took about 30–35 min per participant.

### Data analysis

Prior to statistical data processing, data sets were checked for normality of distribution with the Shapiro-Wilk test. For non-normally distributed data, in addition to means and SDs, Mdns and 95% CIs are reported. Inferential data processing was performed by mixed-model analyses of variance, ANOVA, and post-hoc pairwise comparisons with the JMP software package (version 16.2, SAS Institute, Cary, North Carolina, USA). Non-parametric statistics (Mann–Whitney test and Wilcoxon signed-rank test) was performed for between-group and within-group comparisons, respectively, using MATLAB (version 2023a; MathWorks Inc., Natick, MA, USA).

## Results

### Recognition accuracy

The individual accuracy rates were submitted to a two-way mixed-model ANOVA with the between-subject factor Disorder (Yes/No) and within-subject factor Task (RMET-M/EMF). As expected, a main effect of Disorder was highly significant (*F*(1,54) = 86.99, *p* < 0.001; effect size, *eta-squared η*^*2*^ = 0.62), with SZ patients being less accurate than TD controls. A main effect of Task only tended to reach significance (*F*(1,54) = 3.67, *p* = 0.061; n.s., *η*^*2*^ = 0.06). A Disorder by Task interaction was not significant (*F*(1,54) = 0.40, *p* = 0.531; n.s., *η*^2^ = 0.01).

**Fig. 2:**
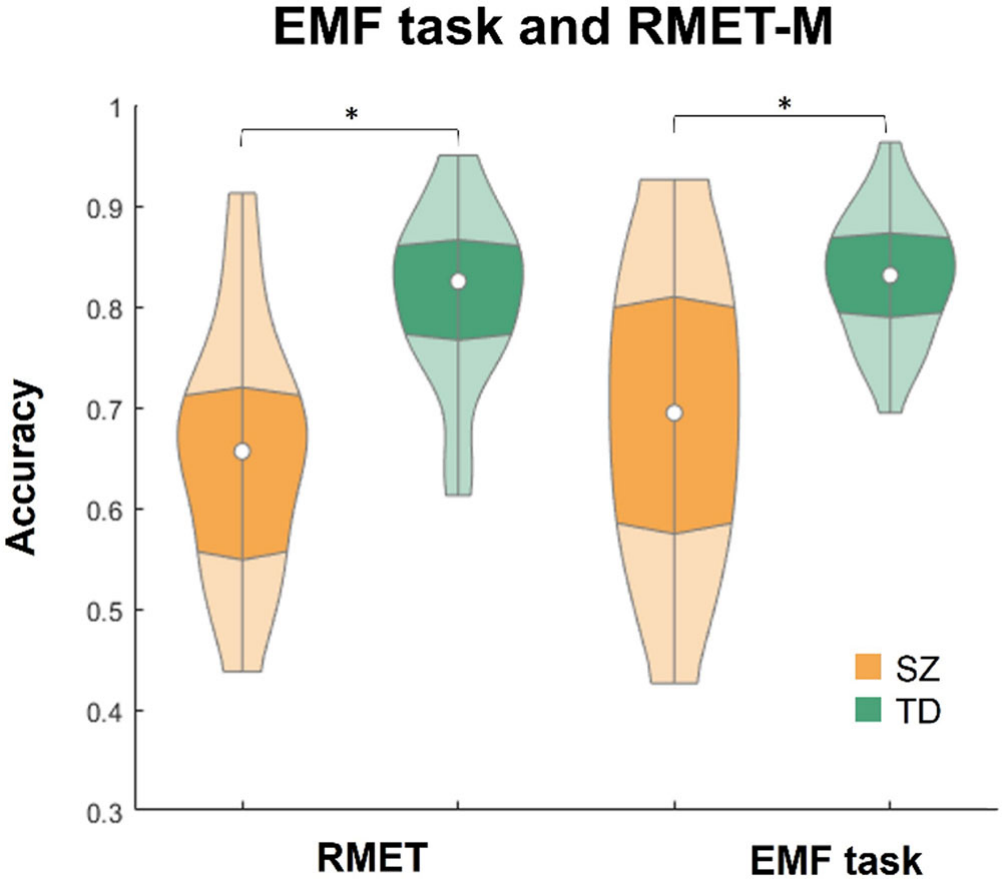
Violin plots of recognition accuracy on the RMET-M and EMF task. The plots are given for SZ (apricot, left violins in each pair) and TD (ocean wave, right violins in each pair) males. Asterisks indicate significant differences (*p* < 0.05).

For the SZ and TD groups separately, accuracy on the RMET-M did not differ from the EMF task (SZ, *t*(27) = 1.60, *p* = 0.119, n.s., effect size, Cohen’s *d* = 0.30, here and further two-tailed; TD, *t*(27) = 1.06, *p* = 0.295, n.s., *d* = 0.27; Fig. 2). However, on both tasks administered, SZ patients demonstrated less accurate recognition than TD individuals (RMET-M, 0.656 ± 0.122 and 0.809 ± 0.084, for SZ and TD, respectively; *t*(54) = 5.51, *p* < 0.004; here and further false discovery rate [FDR] corrected for multiple comparisons; effect size, Cohen’s *d* = 1.47; EMF task, 0.695 ± 0.135 and 0.829 ± 0.062, for SZ and TD, respectively; *t*(54) = 4.78, *p* < 0.001; *d* = 1.28).

### Recognition accuracy on EMF task

For a better understanding of performance on each task separately, the individual accuracy rates for each emotion on the EMF task were submitted to a two-way mixed-model ANOVA with the between-subject factor Disorder (Yes/No) and within-subject factor Emotion (Anger, Happiness, Neutrality, Sadness, Fear, and Disgust). A main effect of Emotion was highly significant (*F*(5,270) = 52.69, *p* < 0.001; *η*^*2*^ = 0.49), as was a main effect of Disorder (*F*(1,270) = 81.36, *p* < 0.001; *η*^*2*^ = 0.60) indicating on overall worse emotion recognition in SZ patients. A Disorder by Emotion interaction was not significant (*F*(5,270) = 1.40, *p* = 0.225, n.s., *η*^*2*^ = 0.03), which indicated that SZ patients were to a comparable degree less accurate than TD individuals on all emotions.

**Fig. 3:**
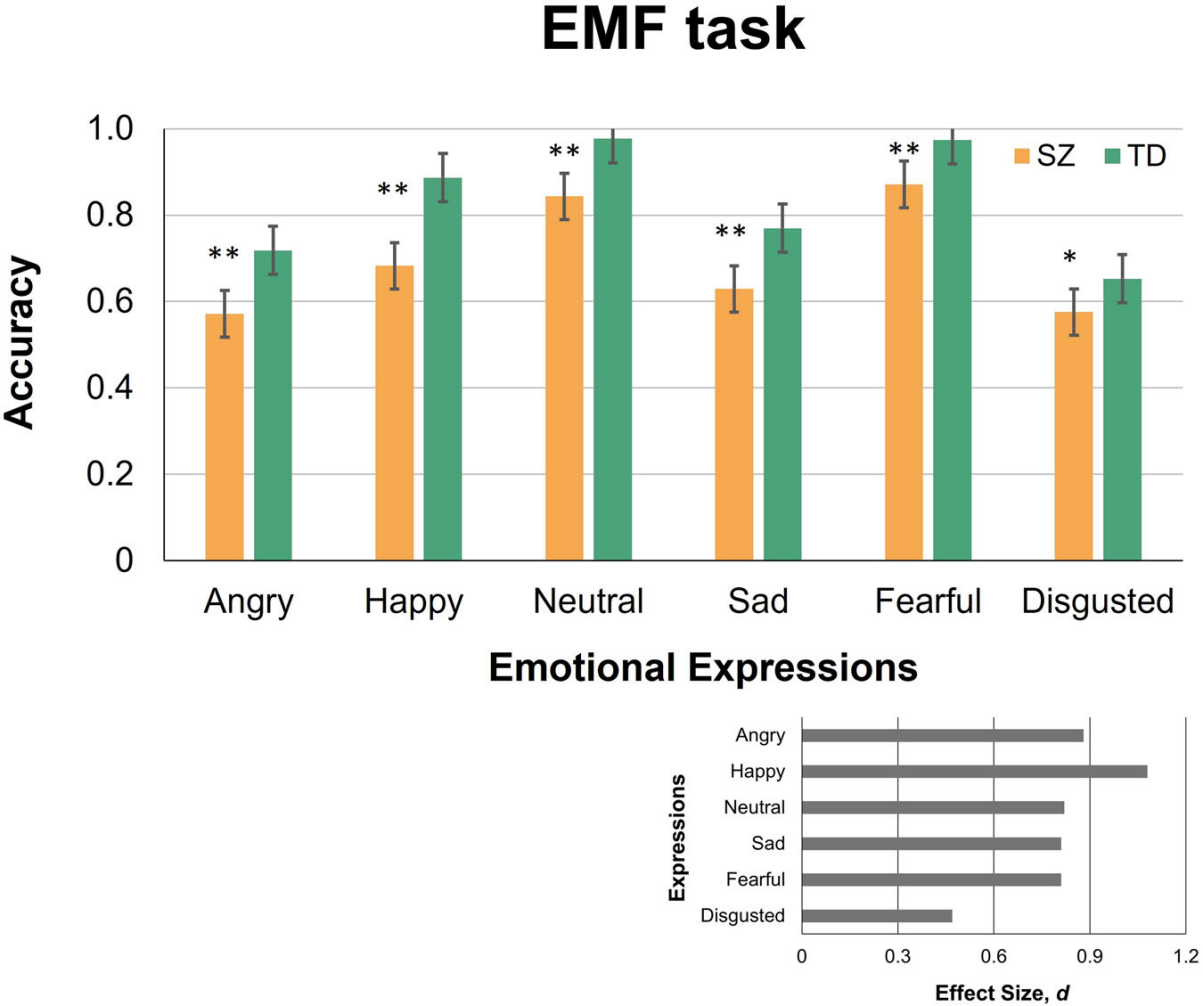
Recognition accuracy of emotions in masked faces (EMF) task. The plots are given for SZ (apricot, left bars in each pair) and TD (ocean wave, right bars in each pair) males. Vertical bars represent ± SEM. Double asterisks indicate significant differences (*p* = 0.006), and a single asterisk a tendency (*p* = 0.08). Effects sizes for comparisons between TD and SZ participants are shown in inset (right bottom plot).

Post-hoc pairwise comparisons of accuracy for each emotion indicated a general impairment of male SZ patients in inferring emotions behind a mask (Fig. 3). For five out of six emotions, SZ patients were less accurate than their TD peers: for anger (SZ, 0.571 ± 0.195; TD, 0.718 ± 0.132; *t*(54) = 3.31, *p* = 0.006, here and further FDR corrected for multiplicity and two-tailed; *d* = 0.88), happiness (SZ, 0.683 ± 0.250, Mdn, 0.722, 95% CI [0.586; 0.78]; TD, 0.887 ± 0.126, Mdn, 0.889, 95% CI [0.838; 0.963]; *U* = 175, *p* = 0.006; *d* = 1.08), neutral expression (SZ, 0.843 ± 0.199, Mdn, 0.944, 95% CI [0.766; 0.92]; TD, 0.977 ± 0.042, Mdn, 1.00, 95% CI [0.961; 0.993]; *U* = 218.5, *p* = 0.006; *d* = 0.82), sadness (SZ, 0.629 ± 0.185; TD, 0.770 ± 0.177, Mdn, 0.778, 95% CI [0.701;0.839]; *U* = 220.5, *p* = 0.006, *d* = 0.81) and fear (SZ, 0.871 ± 0.150, Mdn, 0.917, 95% CI [0.813; 0.929]; TD, 0.974 ± 0.041, Mdn, 1.00, 95% CI [0.958;0.990]; *U* = 221.5, *p* = 0.006; *d* = 0.81). The sole emotion that only tended to reach a significant difference between the groups was disgust (SZ, 0.575 ± 0.191; TD, 0.653 ± 0.131; *t*(54) = 1.77, *p* = 0.083, n.s., *d* = 0.47, corrected for multiple comparisons, two-tailed) most likely because TD individuals also experienced pronounced difficulties in disgust recognition.

As seen in Fig. 3 and indicated by the Steel-Dwass test (Table S2, Supplementary Material), both SZ patients and TD controls exhibited a similar pattern for inferring emotions, with fear and neutral expressions recognized close to the ceiling level and happiness recognized less accurately. Anger, sadness, and disgust were the least recognizable in both groups.

### Recognition accuracy on RMET-M

**Fig. 4:**
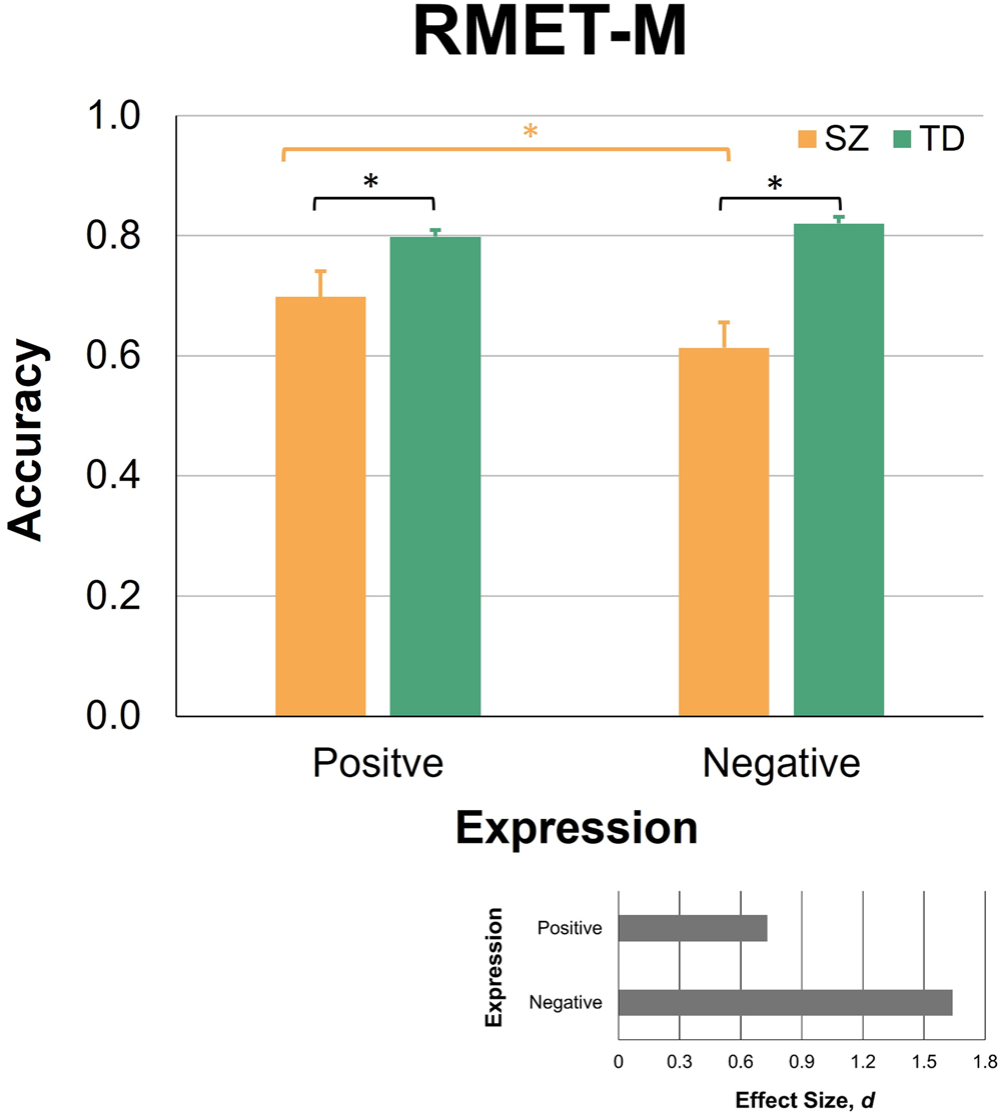
Accuracy rates for positive and negative expressions on the RMET-M in SZ (apricot, left bars in each pair) and TD males (ocean wave, right bars in each pair). Vertical bars represent ± SEM. Asterisks indicate significant differences (*p* < 0.05). Effects sizes for comparisons between TD and SZ participants are shown in inset (right bottom plot).

As with the EMF task, individual accuracy rates for expressions with positive and negative valence on the RMET-M were submitted to a two-way mixed-model ANOVA with the between-subject factor Disorder (Yes/No) and within-subject factor Valence (Positive/Negative). A main effect of Disorder was highly significant (*F*(1,54) = 47.40, *p* < 0.001; *η*^*2*^ = 0.47), indicating generally worse recognition in SZ patients. A main effect of Valence was not significant (*F*(1,54) = 1.96, *p* = 0.167; n.s., *η*^*2*^ = 0.04). A Disorder by Valence interaction was significant (*F*(1,54) = 5.77, *p* = 0.020, *η*^*2*^ = 0.10), which indicated that SZ patients were to a different degree less accurate than TD individuals on images with positive and negative valence (Fig. 4).

Pairwise comparisons showed that as compared with their TD peers, SZ patients were impaired on both recognition of positive (SZ, 0.698 ± 0.158; TD, 0.798 ± 0.121, Mdn, 0.80, 95% CI [0.751;0.845]; *U* = 235.5, *p* = 0.010; here and further FDR corrected for multiple comparisons and two-tailed; effect size, *d* = 0.73) as well as negative expressions (SZ, 0.613 ± 0.123; TD, 0.821 ± 0.129; *t*(54) = 6.15, *p* = 0.003; *d* = 1.64). SZ patients were more accurate in recognition of images with positive than negative valence (*t*(27) = 3.11, *p* = 0.006, *d* = 0.59). By contrast, no difference in recognition accuracy of positive and negative items was found in TD controls (Wilcoxon signed-rank test, *z* = 0.484, *p* = 0.631; n.s., *d* = 0.18).

### Response time

A detailed analysis of response time (RT) is provided in Supplementary Material. The main outcome is that SZ patients are slower than their TD peers in responding on both the RMET-M (SZ, 2.423 ± 0.758, here and further in seconds; TD, 1.876 ± 0.513, Mdn, 1.747, 95% CI [1.669; 2.083]; *U* = 192, *p* = 0.001, here and further FDR corrected for multiple comparisons and two-tailed; *d* = 0.97) and EMF task (1.617 ± 0.417 and 1.169 ± 0.290, for SZ and TD, respectively; *t*(54) = 4.67, *p* = 0.001, *d* = 1.25). Both patients with SZ and TD controls responded faster on the EMF task (SZ, *t*(27) = 6.27, *p* < 0.001, *d* = 1.24; TD, *z* = 4.55, *p* < 0.001, *d* = 3.37).

### Link between EMF task and RMET-M

**Fig. 5:**
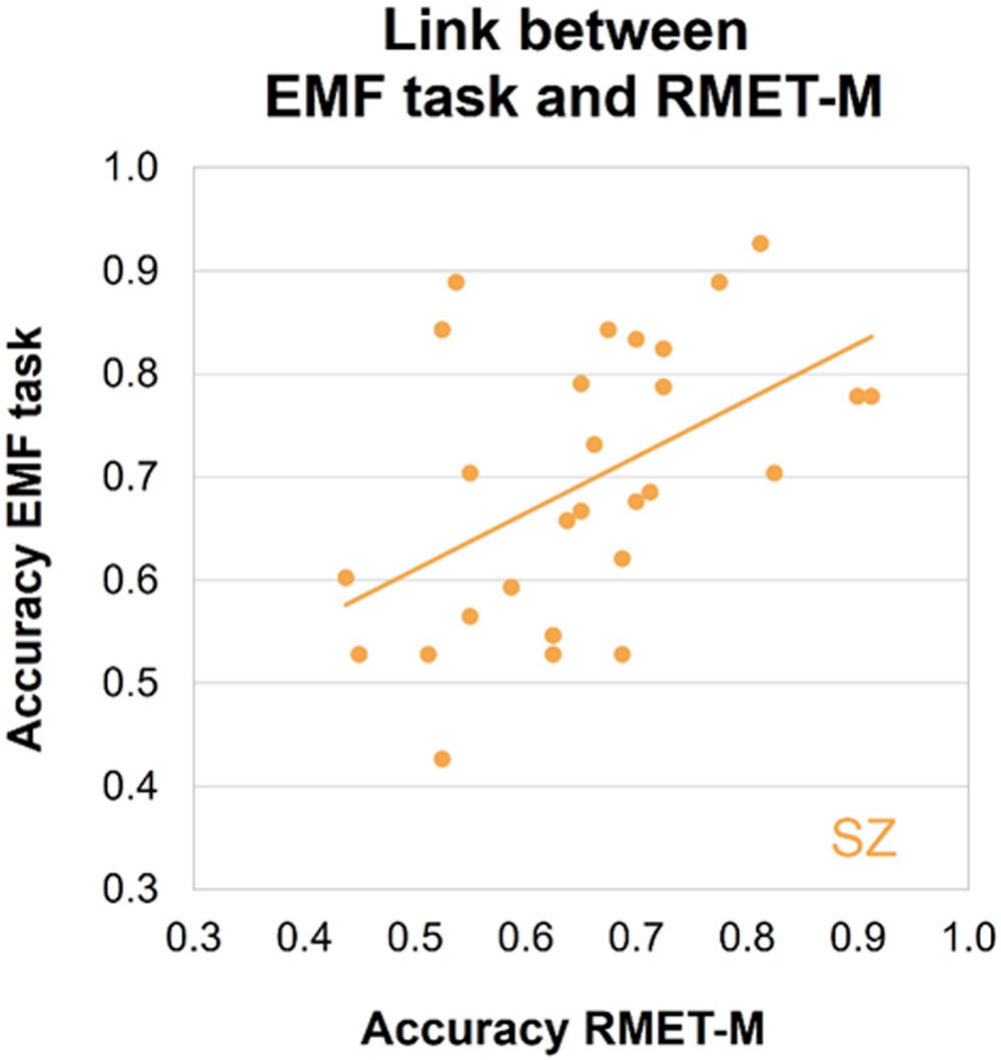
Link between recognition accuracy on the EMF task and RMET-M in SZ patients. Significant positive linear Pearson correlation (*p* = 0.008) was found.

As faces covered by masks contain a comparable amount of information as RMET images^36,46^, we expected to find a correlation between performance on both tasks in SZ patients and TD controls. In SZ individuals, a positive correlation was found between recognition accuracy on the RMET-M and EMF task (Pearson’s product moment correlation, *r*(27) = 0.494, *p* = 0.008; Fig. 5). No correlation in recognition accuracy between the two tasks was found in TD individuals (*r*(27) = 0.094, *p* = 0.631; n.s.; Figure S1, Supplementary Material). For both SZ patients (*r*(27) = 0.451, *p* = 0.016) and their TD peers (*r*(27) = 0.656, *p* < 0.001), RT on the RMET-M positively correlated to RT on the EMF task (Figure S2, Supplementary Material).

In SZ individuals, no link occurred between chronological age and accuracy on the RMET-M (Spearman’s rho, *ρ*(27) = −0.024, *p* = 0.905, n.s.) and EMF task (*ρ*(27) = −0.150, *p* = 0.459, n.s.). TD controls showed a negative correlation between age and accuracy on the EMF task (*ρ*(27)= −0.379, *p* = 0.047), but not on the RMET-M (*ρ*(27)= −0.233, *p* =0.234, n.s.).

## Discussion

The present work was aimed at investigation of reading language of the eyes in male SZ. The findings indicate that (i) patients with SZ are generally less accurate than their TD peers in inferring emotions when only visual information from the eyes is available, as well as mental states of others as assessed by the RMET-M. (ii) In SZ, the emotion recognition profile is similar to TD, with a more accurate recognition of some emotions such as fear, neutral expressions, and happiness than the others (sadness and disgust). However, in SZ, this profile is shifted down: all emotions are recognized less accurately than in TD. On the RMET-M, SZ individuals are also less precise (not only in general, but also separately on the items with positive and negative valence), albeit they perform better on positive than on negative items. (iii) In SZ, accuracy and processing speed on the RMET-M and EMF task positively correlate with each other. Overall, SZ male patients appear to exhibit global deficits in inferring social signals through the eyes.

### Recognition of emotions through the eyes in male schizophrenia

The findings are consistent with previous work suggesting that in typical development, face masks disproportionally hinder emotion recognition leaving inferring fear and neutral expressions almost unharmed, but heavily affecting sadness and disgust (see Introduction; for review^46^). For the first time, however, the study reveals that in SZ, reading basic emotions in the eyes shows the similar uneven profile as in typical development, with still rather well-recognizable fear and neutral expressions and poorly detectable sadness, anger, and disgust. In SZ, the recognition level is substantially lower than in TD for all emotions, suggesting a global deficit in inferring emotions through the eyes instead of selective impairments in recognition of specific emotions.

By using the same methodology, it was recently found that females with MDD demonstrate selective difficulties in inferring basic emotions through the eyes^68^. When only the eyes are visible, inferring anger and, in particular, sadness is less precise in female MDD as compared with typical development, whereas the recognition of fear, happiness, and neutral expressions remains at the same rather high level as in TD individuals. By contrast, the present study reveals that in male SZ, inferring all basic emotions (anger, happiness, neutral expressions, sadness, and fear) is generally poorer, with disgust recognition tending to be less accurate. One plausible account for the lack of a significant difference in disgust recognition may be that even in TD, accuracy is relatively low. The possible sources of difficulties in disgust recognition are discussed earlier^68,71^.

Individuals with SZ are well-known to experience snags in facial affect recognition even in full-seen faces^32,83–85^. However, no consensus has been reached so far in favor of selectivity/specificity (deficits only in distinct emotions or valence) versus globality/generality (deficits in all emotions, either proportional or uneven) of difficulties in emotion recognition^86^. Some studies advocate a global deficit, in particular, in recognition of all negative emotions, and consider this impairment as a vulnerability indicator^87^, trait marker (in particular, for threat recognition^88^), or heritable endophenotype^89^. Other work points to rather selective impairments in inferring fear and anger^90^, or surprise, contempt, sadness, disgust, and neutral expressions (but not happiness, anger, and fear)^31^, or happiness and surprise^91^. This inconsistent outcome most likely may be ascribed to methodological reasons and inhomogeneity of samples. In our view, a general/global or selective deficit in emotion recognition may correspond to a gender-specific profile in social cognition, namely, a global deficit may be attributed primarily to male SZ, whereas a selective deficit to female SZ. However, this assumption requires experimental verification. An alternative explanation for the data controversy may be that a global deficit arises when either visual input is limited (usually, visual cues are redundant to provide efficient social cognition preventing maladaptive behavior) or the task is too demanding.

All in all, the present findings dovetail well with previous work in SZ examining reading faces and bodies. Similar to this study, a global impairment is reported for reading body language (inferring basic emotions in point-light biological motion displays) in SZ individuals (*N* = 84; 53 males)^26^. Patients were not selectively impaired on specific emotions. Instead, they were less accurate than healthy controls on all emotional expressions, including happiness and neutral expressions. Moreover, inferring emotions from body motion exhibits a similar profile in both TD and SZ individuals, with happiness and neutral expressions recognized better than other (*negative*) emotions such as anger and fear (Fig. 1 in^26^).

Reading point-light dynamic faces in SZ^30^ follows in footsteps of reading point-light body motion. As compared to TD peers, inferring facial affect in SZ patients (*N* = 16, 12 males) is proportionally less precise for all emotions (anger, happiness, surprise, sadness, fear, and disgust). Again, the profile of emotion recognition was uneven but rather similar in both SZ and TD individuals, with some (*negative*) emotions (such as disgust, anger, and fear) being less accurately detectable than others such as surprise or happiness.

In a nutshell, SZ patients exhibit a global/generalized deficit in reading emotions in the static eyes as well as in dynamic point-light faces and bodies. For reading language of the eyes, point-light faces and bodies, the recognition pattern in both SZ and TD is uneven and quite similar. The question arises: whether, and, if so, how the flattening/blunting in emotion recognition in SZ is related to flattening in emotion expression? This issue requires further experimental clarification. It is equally important to elucidate the origins of a global impairment in emotion recognition in SZ: whether the proportional decrease in emotion recognition accuracy reflects the specificity of nonverbal social cognition at large or rather stems from other non-social cognitive deficits constituting the very nature of SZ.

### RMET-M in male schizophrenia

The present outcome demonstrates that patients with SZ experience more troubles than their TD peers in inferring mental states (both positive and negative) through the eyes, as assessed by the RMET-M. This outcome is consistent with previous studies^17,40,42,43,92–94^. Furthermore, the deficit occurs already at the early stages of the disorder, as no differences are found between chronic patients and individuals with a first episode of psychosis^13,38^. In chronic SZ, poorer performance is reported in drug-naïve patients compared with those on regular medication^92^ and in symptomatic SZ compared with those in remission^94^.

The present findings show that in SZ, recognition of expressions with positive valence is more accurate than with negative ones. By contrast, females with MDD (examined with the same RMET-M) are less accurate in inferring positive as compared to negative items^68^. In other words, while inferring negative expressions is more strongly impaired in SZ, inferring positive expressions is more challenging for MDD patients. This outcome offers novel insights into the profiles of social cognition deficits in mental disorders that differ in their gender prevalence.

### Relationship between RMET-M and EMF task

As the RMET-M and EMF task contain similar information from the eyes, we expected to find a link in performance between the two tasks. However, a positive correlation in recognition accuracy occurred in SZ male patients, but not in TD controls. By contrast, MDD females (as well as TD controls) did not exhibit any correlation in recognition accuracy^68^. Yet, for all groups (males with SZ, TD male controls, females with MDD, and TD female controls), processing speed of the reading language of the eyes as assessed by both the RMET-M and EMF task were closely related to each other.

A link of RTs (processing speed) between the two tasks suggests a commonality primarily in encoding of the visual input and accumulation of sensory evidence. According to current drift diffusion models of decision making^95,96^, *non-decision* processing time (time needed to process encoded sensory information and to execute a motor response) is critical to account for individual RT variability. In the same vein, a lack of association between two tasks in their accuracy suggests *distinct* latent neurocognitive mechanisms underwriting both tasks’ performance, such as accumulation of sensory evidence for decision making, decision thresholds and criteria. From this standpoint, it appears that (as expected) all groups exhibiting a positive correlation in processing speed between the tasks rely on rather similar visual cues when reading language of the eyes. However, as mentioned earlier^68^, although the visual input is comparable, the tasks fundamentally differ in their origin: inferring basic emotions may be less demanding than reading more sophisticated mental states. This results in the lack of correlation in recognition accuracy in all groups except for male patients with SZ. For SZ patients, both tasks may be challenging in terms of latent neurocognitive mechanisms and decision making that is reflected in the tight link in recognition accuracy between them.

## RÉSUMÉ

The outcome indicates that male individuals with SZ exhibit global deficits in reading language of the eyes as assessed by the RMET-M and in inferring basic emotions through the eyes in faces covered by masks. Moreover, in male SZ, both tasks are tightly linked in terms of recognition accuracy and processing speed. The profile of emotion recognition through the eyes in SZ is not only uneven, but quite similar to typical development, with some emotions being inferred disproportionally better than others. However, males with SZ infer all emotions with lower accuracy. By contrast, in female depression, reading language of the eyes is selectively affected: as compared with controls, patients are profoundly impaired on recognition of expressions with positive (but not negative) valence on the RMET-M, and experience much trouble inferring some basic emotions such as sadness and anger (but not fear, neutral expressions, and happiness) through the eyes^68^. This work offers novel insights into the profile of social cognition deficits in mental disorders that differ in their gender prevalence. For a better understanding of reading language of the eyes in SZ, further research in female SZ is desirable.

Considering the impact of the COVID-19 pandemic^46^, the present findings may contribute to intervention and remediation of inefficient social interaction in male SZ. The findings are valuable also beyond the pandemic, as face masks are of universal use in some professional settings such as medical practice, where they are known to negatively impact patient-healthcare provider interaction^65,71,97–99^. The present study informs that male individuals with SZ require special efforts to reach an optimal level of social interaction based on inferring social signals through the eyes.

## Supplementary information

Supplementary Material

## Data Availability

Data is provided within the manuscript or supplementary information files.
