# Supplementary material for: Inferring social signals from the eyes in male schizophrenia
Source: Schizophrenia (Heidelb). 2024 Nov 14;10(1):107. doi: 10.1038/s41537-024-00527-4 (PMC11564648; doi:10.1038/s41537-024-00527-4)
Supplement: Supplementary file 1 — Supplementary Material [file 41537_2024_527_MOESM1_ESM.pdf]

SUPPLEMENTARY MATERIAL

Inferring social signals through the eyes in male schizophrenia

Annika Resch[1], Jonas Moosavi[1], Alexander N. Sokolov[1], Patrick Steinwand[1], Erika Wagner[1], Andreas J. Fallgatter[1,2], Marina A. Pavlova[1,*]

[1] Department of Psychiatry and Psychotherapy, Tübingen Center for Mental Health (TüCMH), Medical School and University Hospital, Eberhard Karls University of Tübingen, Tübingen, Germany

[2] German Center for Mental Health (DZPG), partner site Tübingen, Germany

SUPPLEMENTARY PARTICIPANT INFORMATION

Twenty-seven out of 28 SZ patients were under medication. Most of them received atypical antipsychotics such as olanzapin (13), aripiprazol (6), risperidon (5), clozapin (4), quetiapin (3), amisulprid (2), sulpirid (1); and/or typical antipsychotics such as haloperidol (3), perazin (1), pipamperon (2); and/or antidepressants [mirazapin (3), sertralin (2), escitalopram (2), venlafaxin (2), citalopram (1), duloxetin (1); lithium (4)]; benzodiazepines such as lorazepam (5); and anticonvulsants such as carbamazepin (1).

SUPPLEMENTARY RESULTS

Recognition accuracy on EMF task

Detailed analysis of the single emotions in the EMF task reveals that SZ patients are significantly impaired in recognition of five out of six emotions: for anger (SZ, 0.571 ± 0.195; TD, 0.718 ± 0.132; $t(54) = 3.31$, $p = 0.006$, here and further FDR corrected for multiplicity and two-tailed; $d = 0.88$), happiness (SZ, 0.683 ± 0.250, Mdn, 0.722, 95% CI [0.586; 0.78]; TD, 0.887 ±0.126, Mdn, 0.889, 95% CI [0.838; 0.963]; U =175, $p = 0.006$; d =1.08), neutral expression (SZ, 0.843 ± 0.199, Mdn, 0.944, 95% CI [0.766; 0.92]; TD, 0.977 ± 0.042, Mdn, 1.00, 95% CI [0.961; 0.993]; $U = 218.5$, $p = 0.006$; $d = 0.82$), sadness (SZ, 0.629 ± 0.185; TD,

0.770 ± 0.177, Mdn, 0.778, 95% CI [0.701;0.839]; *U* = 220.5, *p* = 0.006, *d* = 0.81) and fear (SZ, 0.871 ± 0.150, Mdn, 0.917, 95% CI [0.813; 0.929]; TD, 0.974 ± 0.041, Mdn, 1.00, 95% CI [0.958;0.990]; *U* = 221.5, *p* = 0.006; *d* = 0.81).

No link between recognition accuracy on EMF task and RMET-M in TD controls

As indicated in the main text, by contrast with patients with SZ, no correlation in recognition accuracy between the two tasks was found in TD individuals (*r*(27) = 0.094, *p* = 0.631; n.s.; **Figure S1**).

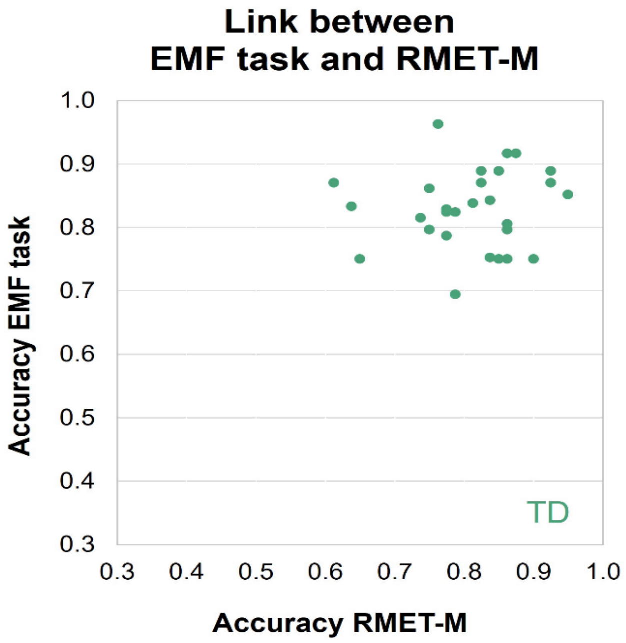

Figure S1. No correlation occurred between recognition accuracy on the RMET-M and EMF task in TD controls.

Response Time

The individual response time (RT) for correct responses on the RMET-M and EMF task was submitted to a two-way mixed-model ANOVA with the within-subject factor Task (RMET-M/EMF) and between-subject factor Disorder (Yes/No). A highly significant main effect of Disorder was found ($F(1,54) = 43.70$, $p < 0.001$; effect size, *eta-squared* $\eta^2 = 0.45$), with TD

controls responding faster than individuals with SZ. A main effect of Task was also significant ($F(1,54) = 100.88$, $p < 0.001$; $\eta^2 = 0.65$), with generally faster responses on the EMF task. A Disorder by Task interaction was not significant ($F(1,54) = 0.44$, $p = 0.513$; n.s., $\eta^2 = 0.01$).

Response time on EMF task

To examine differences in RT on each task separately, the individual RT for correct responses on the EMF task was submitted to a two-way mixed model ANOVA with the within-subject factor Emotion (Angry, Happy, Neutral, Sad, Fearful, Disgust) and the between-subject factor Disorder (Yes/No). A main effect of Disorder was highly significant ($F(1,270) = 256.37$, $p<0.001$, $\eta^2 = 0.83$) with patients responding generally slower than TD individuals. A main effect of Emotion was also highly significant ($F(5,270) = 48.47$, $p < 0.001$, $\eta^2 = 0.47$). A Disorder by Emotion interaction was not significant ($F(5,270) = 1.18$, $p = 0.317$; n.s., $\eta^2 = 0.02$), which indicates that patients with SZ were to a similar degree slower than TD controls on recognition of all presented emotions.

Post-hoc pairwise comparisons show that males with SZ responded slower to all emotions: anger (SZ, 1.693 ± 0.505; TD, 1.234 ± 0.396, Mdn. 1.112, 95% CI [1.084; 1.403]; $U = 193$, $p = 0.001$, here and further FDR corrected and two-tailed; $d = 0.97$), happiness (SZ, 1.363 ± 0.463; TD, 0.871 ± 0.238; $t(54) = 4.99$, $p < 0.001$; $d = 1.33$), neutral expression (SZ, 1.408 ± 0.444; TD, 0.919 ± 0.295, Mdn 0.832, 95% CI [0.799; 1.038]; $U = 140$, $p < 0.001$; $d = 1.32$), sadness (SZ, 1.883 ± 0.574; TD, 1.580 ± 0.336; $t(54) = 2.41$, $p = 0.019$; $d = 0.65$), fear (SZ, 1.689 ± 0.486, Mdn 1.616, 95% CI [1.492;1.885]; TD, 1.217 ± 0.391; $U = 176$, $p < 0.001$; $d = 1.07$), and disgust (SZ, 1.849 ± 0.553; TD, 1.344 ± 0.438; $t(54) = 3.79$, $p < 0.001$; $d = 1.01$).

Response time on RMET-M

The individual RT for correct responses for positive and negative expressions on the RMET-M was submitted to a two-way mixed-model ANOVA with the between-subject factor Disorder

(Yes/No) and within-subject factor Expression (Positive/Negative). A main effect of Disorder was highly significant ($F(1,54) = 144.48$, $p < 0.001$; $\eta^2 = 0.73$), with patients responding generally slower than TD individuals. A main effect of Expression was also significant ($F(1,54) = 10.95$, $p = 0.002$; $\eta^2 = 0.17$), with generally slower responses on negative items. A Disorder by Expression interaction was not significant ($F(1,54) = 0.35$, $p = 0.559$; n.s., $\eta^2 = 0.01$).

Patients with SZ were slower compared to TD controls for both positive (SZ, 2.374 ± 0.777; TD, 1.793 ± 0.548, Mdn, 1.664 , 95% CI [1.572;2.015]; $U = 194$, p=0.003, here and further FDR corrected and two-tailed; $d = 0.96$) and negative (2.500 ± 0.799 and 1.973 ± 0.522, for SZ and TD, respectively; $t(54) = 2.92$, $p = 0.005$; $d = 0.78$) expressions. In a within-group analysis, TD individuals were faster in response to positive compared to negative expressions (Wilcoxon signed-rank test, $z = 2.94$, $p = 0.005$; $d = 1.34$), whereas no such difference occurred in the SZ group ($t(27) = 1.70$, $p = 0.101$; n.s., $d = 0.16$).

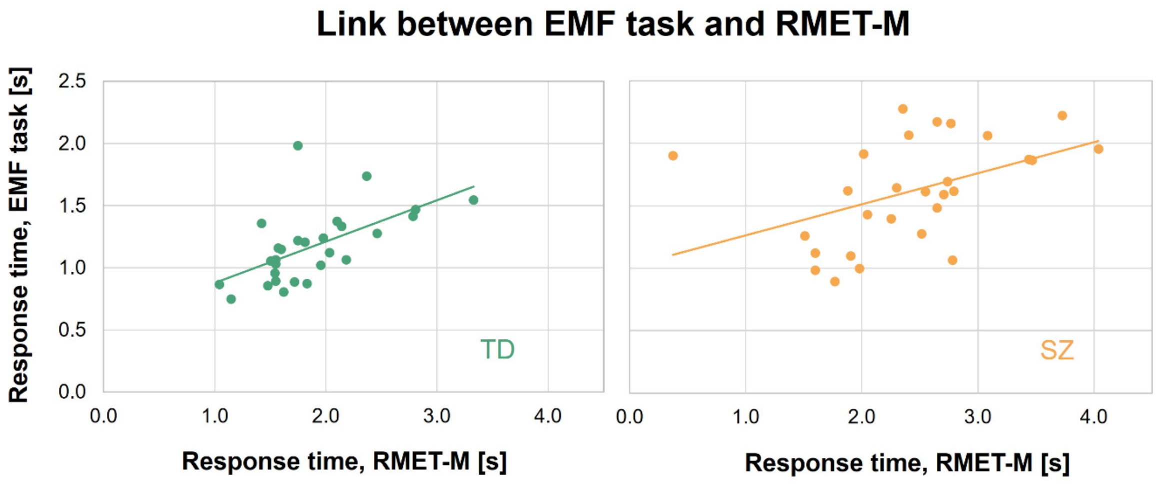

Figure S2. Relationship between RT on the EMF task and RMET-M in TD controls ($p < 0.001$; ocean wave, left panel) and SZ patients ($p = 0.016$; apricot, right panel).

Link in RT between EMF task and RMET-M

For both SZ patients (Pearson product moment correlation, $r(27) = 0.451$, $p = 0.016$) and their TD peers ($r(27) = 0.656$, $p = 0.002$), RT on the RMET-M positively correlated to RT on the EMF task (**Figure S2**).

Neither in SZ patients nor in TD controls was there a link between chronological age and RT on the RMET-M (SZ, Spearman's *rho*, $\rho(27) = 0.233$, $p = 0.233$; n.s.; TD, $\rho(27) = 0.094$, $p = 0.636$; n.s.) and on the EMF task (SZ, $\rho(27) = 0.281$, $p = 0.147$; n.s.; TD, $\rho(27) = 0.140$, $p = 0.479$; n.s.).

Table S1. Comorbidity.

	**Comorbidities (ICD-10 Code)**
P01	Postschizophrenic depression (F20.4)<br>Mental and behavioral disorders due to use of tobacco, dependence syndrome (F17.2)
P02	Mental and behavioral disorders due to use of cannabis, dependence syndrome (F12.2)<br>Mental and behavioral disorders due to use of tobacco, dependence syndrome (F17.2)<br>Mental and behavioral disorders due to use of alcohol, harmful use (F10.1)<br>Mental and behavioral disorders due to use of cocaine, harmful use (F14.1)<br>Mental and behavioral disorders due to use of other stimulants, including caffeine, harmful use (F15.1)
P04	Mental and behavioral disorders due to use of tobacco, dependence syndrome (F17.2)
P05	Mental and behavioral disorders due to use of tobacco, dependence syndrome (F17.2)
P06	PTSD/post-traumatic stress disorder (F43.1)
P07	Mixed and other personality disorders (F61)<br>Conduct disorder (F91)<br>Mental and behavioral disorders due to use of tobacco, dependence syndrome (F17.2)
P08	Mental and behavioral disorders due to use of tobacco, dependence syndrome (F17.2)<br>Mental and behavioral disorders due to the use of cannabis, dependence syndrome (F12.2)<br>Mental and behavioral disorders due to use of other stimulants, including caffeine, harmful use (F15.1)
P09	Obsessive compulsive disorder (F42.1)<br>Mild mental retardation (F70.0)
P11	Pervasive developmental disorder (F84.9)<br>Mixed and other personality disorders (F61)
P14	Mental and behavioral disorders due to use of alcohol, dependence syndrome (F10.2)
P15	Mental and behavioral disorders due to use of tobacco, dependence syndrome (F17.2)
P16	Mental and behavioral disorders due to use of tobacco, dependence syndrome (F17.2)
P19	Mental and behavioral disorders due to use of tobacco, dependence syndrome (F17.2)
P20	Recurrent depressive disorder (F33.2)<br>Disturbance of activity and attention (F90.0)
P24	Mental and behavioral disorders due to use of other psychoactive substances, harmful use (F19.1)
P26	Mental and behavioral disorders due to use of cannabis, dependence syndrome (F12.2)<br>Mental and behavioral disorders due to use of other psychoactive substances, harmful use (F19.1)
P27	Mental and behavioral disorders due to use of opioids, dependence syndrome (F11.2)<br>Mental and behavioral disorders due to use of cannabis, dependence syndrome(F12.2)<br>Mental and behavioral disorders due to use of tobacco, dependence syndrome (F17.2)

Table S2. Pairwise Steel-Dwass comparisons for recognition of emotions behind a mask.

Emotion	Linking Letter	Median	Lower 95% CI	Upper 95% CI	*P*
**SZ**					
neutral	A	0.94	0.77	0.92	1.000
fearful	A	0.92	0.81	0.93	0.016
happy	B	0.72	0.59	0.78	0.753
sad	B	0.61	0.70	0.84	0.958
disgusted	B	0.61	0.50	0.65	1.000
angry	B	0.56	0.50	0.65	
**TD**					
neutral	A	1.00	0.96	0.99	1.000
fearful	A	1.00	0.96	0.99	0.014
happy	B	0.89	0.84	0.96	0.075
sad	B	0.78	0.70	0.84	0.559
angry	B	0.72	0.67	0.77	0.418
disgusted	B	0.64	0.60	0.70	

Note: P-values indicate differences in recognition rates for the current and subsequent emotion. Emotions are ordered according to their recognizability from the most to the least recognizable. The same letters link emotions without differences in recognition.

Table S3. Recognition accuracy: group comparisons.

	SZ			TD					
	**Mean ± SD**	**Mdn**	**95% CI**	**Mean ± SD**	**Mdn**	**95% CI**	**Mann-Whitney *U* / *t* - test**	***P* uncorr.**	***P* corr.**
anger	0.57 ± 0.20			0.72 ± 0.13			*t*(54) = 3.31	0.002**	0.006**
happiness	0.68 ± 0.25	0.72	[0.59; 0.78]	0.89 ± 0.13	0.89	[0.84; 0.96]	*U* = 175.0	0.001**	0.006**
neutral	0.84 ± 0.20	0.94	[0.77; 0.92]	0.98 ± 0.04	1.00	[0.96; 0.99]	*U* = 218.5	0.005**	0.006**
sadness	0.63 ± 0.19			0.77 ± 0.18	0.78	[0.70; 0.84]	*U* = 220.5	0.005**	0.006**
fear	0.87 ± 0.15	0.92	[0.81; 0.93]	0.97 ± 0.04	1.00	[0.96; 0.99]	*U* = 221.5	0.005**	0.006**
disgust	0.58 ± 0.19			0.65 ± 0.13			*t*(54) = 1.77	0.083*	0.083*

Note: For non-normally distributed data, additionally to means and SDs, Mdns and 95% CIs are reported.
Double asterisks indicate significant differences ($p < 0.05$), single asterisks indicate a tendency ($0.05 < p < 0.1$).
